# Single-Molecule Atomic Force Microscopy Reveals Clustering of the Yeast Plasma-Membrane Sensor Wsc1

**DOI:** 10.1371/journal.pone.0011104

**Published:** 2010-06-14

**Authors:** Jürgen J. Heinisch, Vincent Dupres, Sabrina Wilk, Arne Jendretzki, Yves F. Dufrêne

**Affiliations:** 1 Fachbereich Biologie/Chemie - AG Genetik, Universität Osnabrück, Osnabrück, Germany; 2 Institute of Condensed Matter and Nanosciences, Université Catholique de Louvain, Louvain-la-Neuve, Belgium; Auburn University, United States of America

## Abstract

Signalling is a key feature of living cells which frequently involves the local clustering of specific proteins in the plasma membrane. How such protein clustering is achieved within membrane microdomains (“rafts”) is an important, yet largely unsolved problem in cell biology. The plasma membrane of yeast cells represents a good model to address this issue, since it features protein domains that are sufficiently large and stable to be observed by fluorescence microscopy. Here, we demonstrate the ability of single-molecule atomic force microscopy to resolve lateral clustering of the cell integrity sensor Wsc1 in living *Saccharomyces cerevisiae* cells. We first localize individual wild-type sensors on the cell surface, revealing that they form clusters of ∼200 nm size. Analyses of three different mutants indicate that the cysteine-rich domain of Wsc1 has a crucial, not yet anticipated function in sensor clustering and signalling. Clustering of Wsc1 is strongly enhanced in deionized water or at elevated temperature, suggesting its relevance in proper stress response. Using *in vivo* GFP-localization, we also find that non-clustering mutant sensors accumulate in the vacuole, indicating that clustering may prevent endocytosis and sensor turnover. This study represents the first *in vivo* single-molecule demonstration for clustering of a transmembrane protein in *S. cerevisiae*. Our findings indicate that in yeast, like in higher eukaryotes, signalling is coupled to the localized enrichment of sensors and receptors within membrane patches.

## Introduction

The evolution of uni- and multicellular organisms has produced a variety of cellular devices which enable cells to react to environmental changes. In mammals, cell surface receptors are frequently triggered by hormones and depend on the oligomerization of the receptor molecules to generate an intracellular signal, a strategy examplified by mammalian receptor tyrosine kinases (see [1], [2] for recent reviews). Notably, the lateral organization of protein complexes within the plasma membrane in specific microdomains enriched in sphingolipids and sterols (“lipid rafts”) [3] is thought to be crucial in a variety of signalling processes governing diverse biological reactions such as endocytosis, cell adhesion, apoptosis or immune responses (reviewed in [4]). Because the putative size of individual lipid rafts in higher eukaryotes is estimated to be in the 20–100 nm range and to be transient, their direct visualization in live cells remains very challenging [5], [6]. In contrast, in the yeast *Saccharomyces cerevisiae*, the unicellular model eukaryote, stable microdomains have been observed within the plasma membrane by the use of specific GFP-labelled marker proteins [7]. These fluorescence studies have shown that a so-called MCP (for “membrane compartment with Pma1”) compartment forms a network-like structure, defined by its constituent proton ATPase Pma1, while the MCC (“membrane compartment with Can1”) compartment forms 300 nm patches and houses a number of proton symporters, as well as a component of the eisosomes [8].

An essential feature of yeast and other fungi is their rigid cell wall, which serves as a first barrier to extracellular stresses [9]. Consequently, a leak in cell wall integrity (CWI) - which can be induced by surface stresses such as low osmolarity or compounds interfering with cell wall polysaccharides - has to be detected and repaired immediately. The proper cellular reaction is ensured by signalling through the so-called CWI pathway [10]. Surface stresses are first detected by a family of five plasma membrane sensors in this pathway, namely Wsc1, Wsc2, Wsc3, Mid2 and Mtl1 [11], [12]. Upon activation, they trigger an intracellular signalling chain, which involves a conserved mitogen activated protein kinase (MAPK) cascade and the transcription factor Rlm1 (reviewed in [13]). A characteristic feature of the Wsc-family of sensors is the presence of a periplasmatic cysteine-rich domain (CRD; also referred to as “WSC domain”) near the amino-terminal end, followed by a highly mannosylated serine/threonine rich region (STR), which confers nanospring properties to the sensor [14]. A complete deletion of the CRD sequence, albeit not investigated for its effect on the overall protein structure, renders Wsc1 non-functional [15]. Although CRD homologous domains have been detected in 86 proteins from viral, bacterial and eukaryotic origins, their exact function remains obscure [16]. Owing to its presence in the yeast Wsc-sensor family and in a fungal exo-glucanase it has been speculated to mediate interactions with carbohydrate moieties. Interestingly, a CRD domain is also present near the amino-terminal end of the mammalian polycystin protein (PKD1). PKD1 is a mechanosensor whose deficiency causes a prominent hereditary disease in humans [17]. Here again, the detailed function of the CRD sequence has not yet been elucidated.

Fluorescence microscopy revealed that Wsc1-GFP proteins reside in membrane patches within the plasma membrane, both in *S. cerevisiae* and its close relative *Kluyveromyces lactis* [18], [19]. However, the structure-function relationships of Wsc1 clustering are currently unknown. In particular, two key questions still need to be answered: i) what is the structural basis for clustering, i.e. which sensor domain is mechanistically implicated in the process? ii) is clustering of biological relevance, particularly in sensing and signalling? Here, we used single-molecule atomic force microscopy (AFM) [20], [21] to reveal function-related clustering of Wsc1 sensors in living *S. cerevisiae* cells. We showed that clustering requires the CRD domain and is stimulated under stress conditions. Combining these single-molecule analyses with GFP-localization studies, we also suggest that the function of Wsc1 is coupled to its local enrichment within membrane patches, for which we propose the term Wsc1 “sensosome”.

## Results

### AFM demonstrates clustering of Wsc1 in live cells

**Figure 1:**
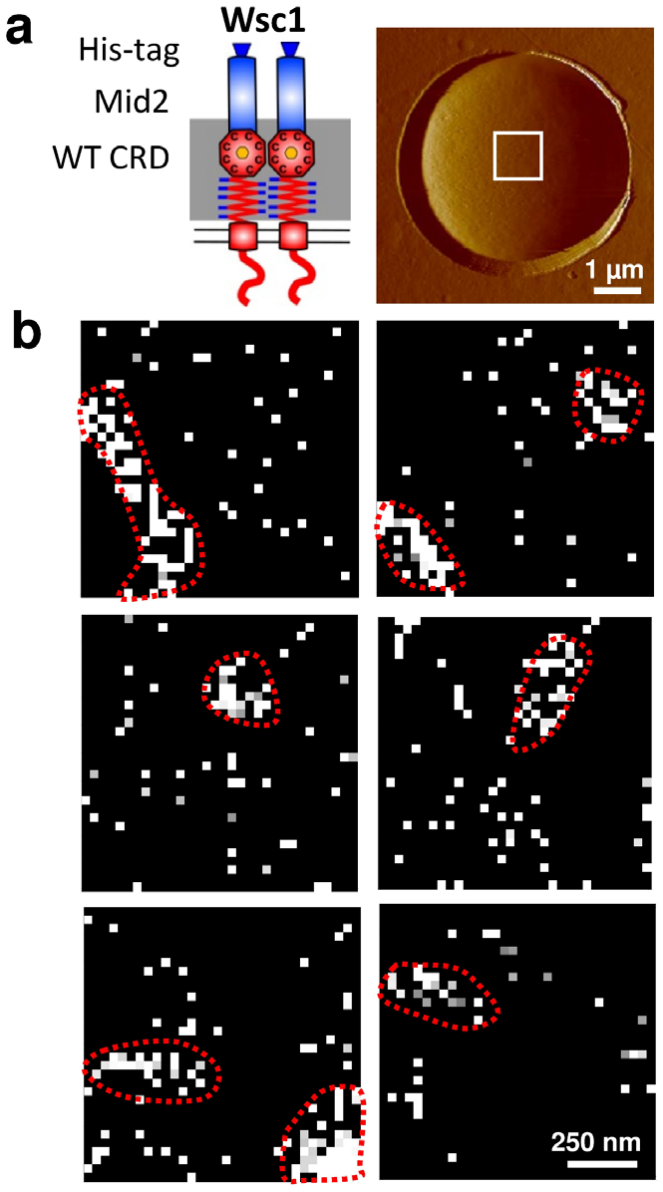
Single-molecule mapping reveals clustering of Wsc1 in live cells. (a) AFM deflection image of a yeast cell trapped into a porous polymer membrane, recorded in buffer solution (sodium acetate + sucrose 100 mM; pH 4.75) at 25°C. As shown in the left cartoon, the cells express elongated, fully functional Wsc1-Mid2 hybrid sensor bearing an His-tag. (b) Representative adhesion force maps obtained by scanning 1 µm×1 µm areas on different cells with a Ni^++^-NTA-tip in buffer solution. The heterogeneous distribution of the bright pixels, which represent the detection of single sensors, clearly documents the formation of nanoscale clusters (highlighted by dotted red lines). We define a cluster as a group of sensors containing at least 10 molecules (bright pixels) either in direct contact with each other or separated by no more than one dark pixel. All maps were obtained using a retraction speed of 1,500 nm s^−1^ corresponding to a loading rate of 9,000 pN s^−1^, and an interaction time of 500 ms. The data shown are representative of results obtained on 12 different cells using 14 different tips.

We used AFM to probe the distribution of Wsc1 sensors on living *S. cerevisiae* cells, with the aim to determine whether they are evenly distributed or clustered (Fig. 1 & Fig. S1). Native sensors are too short to reach the outermost cell surface, thus to be probed by AFM. For our single-molecule analyses, we therefore employed an elongated, fully functional, Wsc1-Mid2 hybrid sensor bearing an His-tag (described in [14]). For this purpose, the respective constructs were introduced on a *CEN/ARS* vector into a recipient strain lacking the endogenous *WSC1* allele (HOD48-1D). Cells producing this modified sensor were trapped into porous polymer membranes and observed using topographic imaging (Fig. 1a). High-resolution images revealed a smooth and homogeneous surface, consistent with earlier AFM analyses [22]. His-tagged sensors were then detected by scanning the cell surface with an AFM tip bearing Ni^2+^-nitriloacetate (NTA) groups. A substantial fraction (7%) of the force curves recorded across the cell surface displayed single adhesion force peaks, the remaining curves exhibiting no adhesion (Fig. S1). The corresponding adhesion force histogram displayed a single maximum with a mean magnitude of 207±54 pN (n = 4096). In the light of the literature data [14], [23], we attribute these forces to the rupture of single NTA-His bonds, thus to the detection of single His-tagged sensors. The specificity of these analyses was confirmed by showing a dramatic reduction of adhesion events in the absence of Ni^2+^, or on related yeast strains lacking the sensors [14].

We next recorded adhesion maps over 1 µm×1 µm areas to resolve the spatial arrangement of the sensors (Fig. 1b). Although some sensor molecules were found to be isolated, many of them were assembled into clusters featuring an area of 0.04±0.01 µm^2^ (n = 22 different clusters from different maps) and an equivalent diameter of 230 nm (calculated assuming a circular shape). This represents the first *in vivo* single-molecule demonstration that a transmembrane protein is clustered in *S. cerevisiae*. Interestingly, we note that the size of Wsc1 clusters is in the range of the 300 nm large patches reported for MCC proteins in *S. cerevisiae*, and clearly larger than the putative size of rafts in higher eukaryotes (<20–100 nm [24]). Thus, unlike membrane domains in mammalian cells, yeast membrane domains such as the one carrying Wsc1 and MCC domains [7], are sufficiently large, distant from each other, and stable to be resolved both by optical and scanning probe microscopy techniques.

### Stress conditions enhance sensor clustering

**Figure 2:**
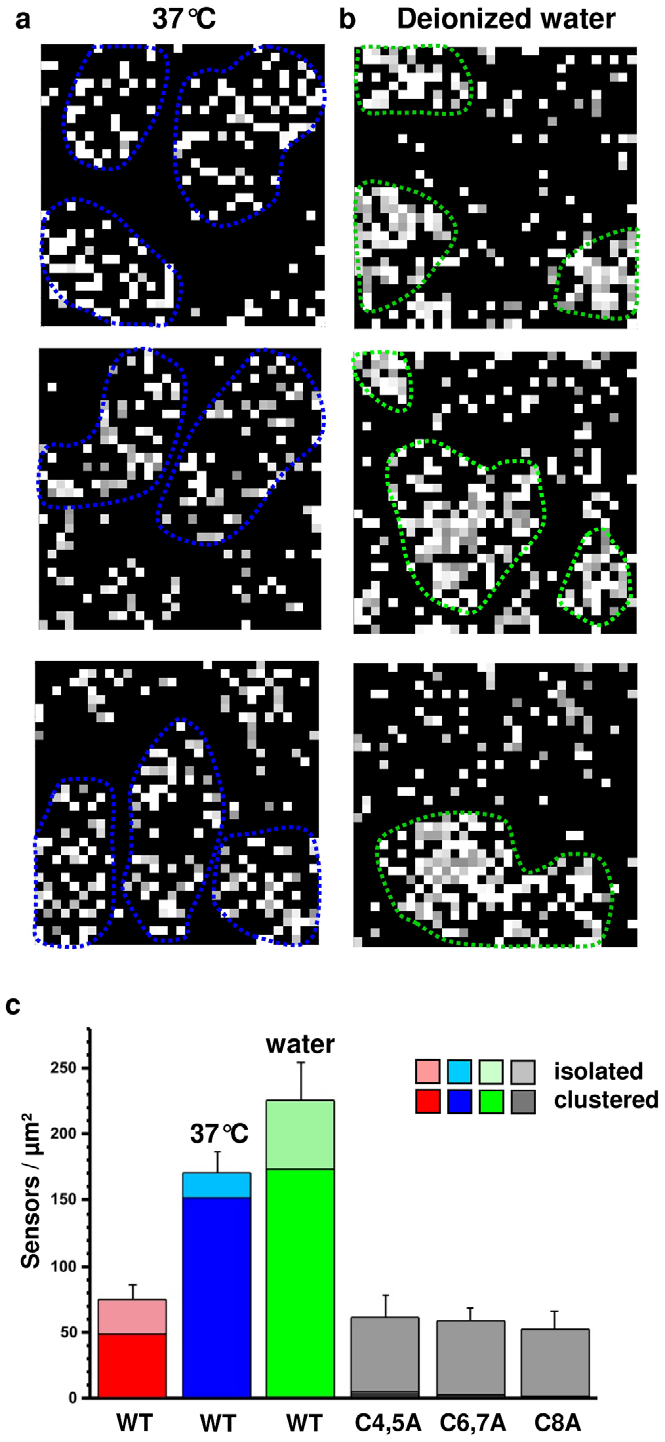
Clustering of Wsc1 is stimulated under stressing conditions. (a, b) Adhesion force maps (1 µm×1 µm) recorded with a Ni^++^-NTA-tip either in buffer solution at 37°C (a, heat shock) or in deionized water at 25°C (b, hypoosmotic shock). Stressing conditions strongly enhance Wsc1 clustering (clusters are highlighted by dotted blue and green lines). For both conditions, the characteristic shape of the force curves confirmed they reflected the detection of single His-tagged sensors. For Fig. 2b, similar data were obtained when deionized water was quickly exchanged by a buffered solution. Here again, we define a cluster as a group of sensors containing at least 10 molecules (bright pixels) either in direct contact with each other or separated by no more than one dark pixel. For each condition, cells were treated for 15 min prior to AFM measurements. The data shown are representative of results obtained on 6 different cells using 6 different tips. (c) Surface density histograms showing the number of sensors per µm^2^ measured (from left to right): for wild-type Wsc1 in buffer at 25°C (n = 16 maps containing 1024 data points each), for wild-type Wsc1 in buffer at 37°C (n = 8 maps) and in deionized water at 25°C (n = 12 maps), as well as for Wsc1_C4,5A_ (n = 7 maps), Wsc1_C6,7A_ (n = 7 maps), and Wsc1_C8A_ mutants (n = 12 maps) in buffer at 25°C. Darker and lighter colors represent the surface density of clustered and isolated sensors.

Is Wsc1 clustering stimulated under stressing conditions? To address this question, we explored the influence of environmental conditions that activate the CWI signalling pathway, i.e. elevated temperature (heat shock) or deionized water (hypoosmotic shock). Fig. 2 shows that both conditions increased the total sensor surface density from 75±22 sensors/µm^2^ (n = 16 maps of 1024 data points recorded over 1 µm^2^) to 171±16 sensors/µm^2^ (elevated temperature, n = 8) and to 225±28 sensors/µm^2^ (deionized water, n = 12). In addition, clustering was strongly enhanced since we observed much larger fractions of clustered sensors (Fig. 2c), as well as a dramatic increase of the mean cluster size, from 0.04±0.01 µm^2^ (n = 22) to 0.15±0.04 µm^2^ (n = 13) and 0.14±0.06 µm^2^ (n = 13), respectively, corresponding to an increase of the equivalent diameter from 230 nm to 440 and 420 nm. Note that in deionized water, quantification of the clusters is rendered more difficult since many sensors were assembled in a complex, heterogeneous pattern rather than forming well-defined clusters.

### The CRD domain of Wsc1 is essential for proper sensor function

**Figure 3:**
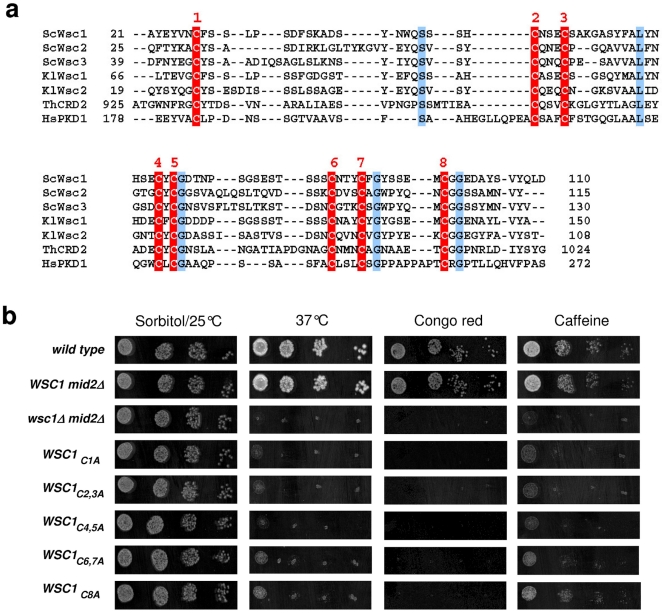
The conserved CRD domain of Wsc1 is essential for proper sensor function. (a) Alignment of the deduced amino acid sequences of selected proteins carrying a CRD domain. The eight conserved cysteine residues are numbered consecutively and highlighted in red. Other conserved amino acid residues identical in all proteins are highlighted in blue. Numbers before and after each sequence refer to the position of the first and last amino acid relative to the N-terminal end (assuming that the starting methionine is not processed). For alignment, the CloneManager Suite programm version 9 was used at standard settings. Wsc-sensor sequences are from Sc  =  *Saccharomyces cerevisiae* and Kl  =  *Kluyveromyces lactis*. ThCRD2  =  homologous sequence from β-1-3 exoglucanase of the fungus *Trichoderma harzianum*. HsPKD1  =  homologous sequence from the human polycystin. For sequence references consult [16], [18]. (b) Serial dilution drop assays. Yeast strains with the indicated alleles were grown overnight into late logarithmic phase, adjusted to OD_600_  = 0.1, and 3 µl each of ten-fold serial dilutions were dropped onto rich medium (YEPD) under stress conditions as indicated. 1 M sorbitol was added for the non-stressed control. Congo red was applied at a concentration of 0.1 mg/ml, caffeine at 7.5 mM. Plates were incubated for 3 days at 25°C, except for the indicated heat stress at 37°C. Scanned images in each column were taken from the same plate, adjusted for brightness and contrast with the CorelDraw photoshop programm. None of the cysteine mutants displayed significant growth under stress conditions as compared to the control strain in the second lane.

The Wsc-family of CWI sensors in yeast is characterized by a cysteine-rich domain (CRD) containing eight conserved cysteine residues which is also found in proteins of other organisms (Fig. 3a), and cannot be deleted without loss of function [15]. For our single-molecule AFM analyses, we employed *in vitro* mutagenesis to substitute the cysteine residues either individually or pairwise for alanines. Thus, five different mutants were constructed (Wsc1_C1A_, Wsc1_C2,3A_, Wsc1_C4,5A_, Wsc1_C6,7A_, Wsc1_C8A_) and first assessed for their *in vivo* function in a strain also lacking the Mid2 sensor (a *mid2* deletion significantly enhances the growth defects of a *wsc1* defective strain, which facilitates its phenotypic characterization [19]). For this purpose, the mutations were introduced individually at the chromosomal *WSC1* locus and the strains obtained were tested in drop dilution assays for their growth under different stress conditions. Note that except for the cysteine mutations Wsc1 remained unaltered in these experiments, i.e. it did not carry the elongation needed for AFM detection employed above. As evident from Fig. 3b, none of the mutants grew at elevated temperature (37°C) or in the presence of Congo red or caffeine, where a *mid2* deletion with a wild-type copy of *WSC1* is not impaired. Complementation studies in a single *wsc1* deletion background gave similar results. This finding indicates that the cysteines are crucial for Wsc1 function. Since no phenotypic differences between the five mutants were observed, we concentrated hereafter mainly on the Wsc1_C4,5A_ mutant, with some complementary studies on the Wsc1_C6,7A_ and the Wsc1_C8A_ mutants.

### Wsc1 clustering is controlled by the CRD domain

**Figure 4:**
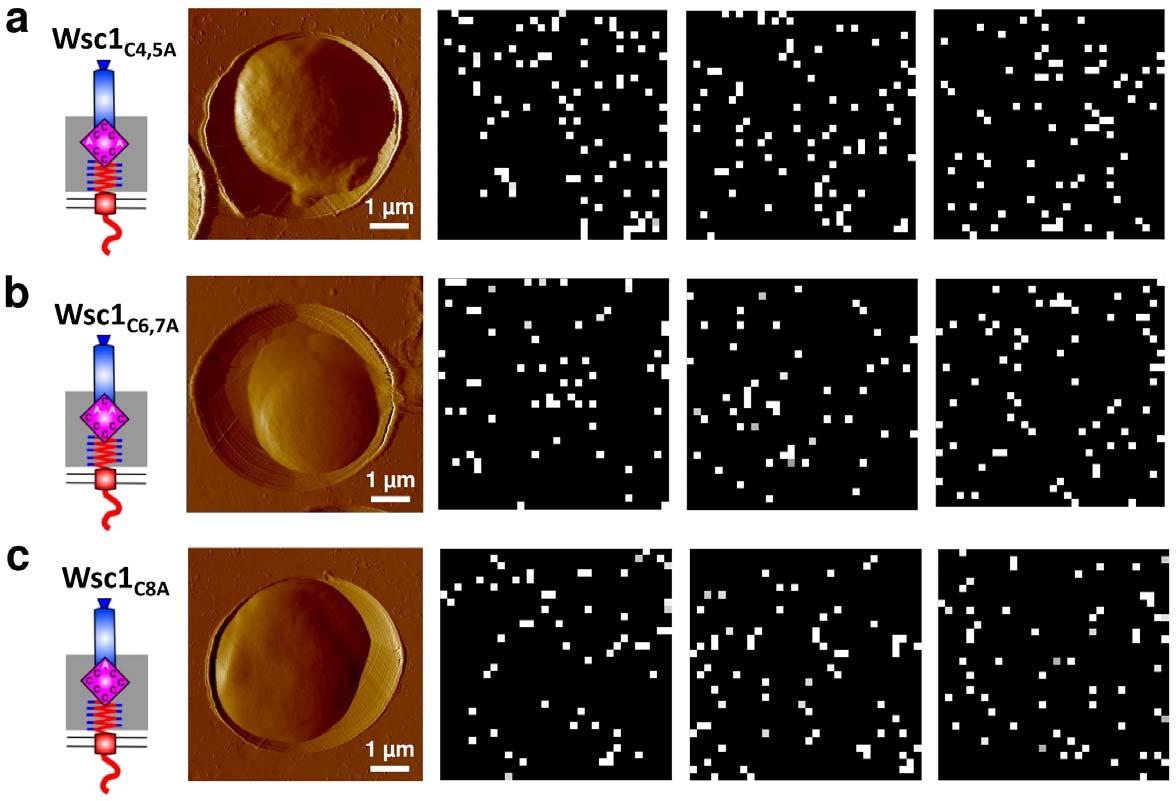
Clustering of Wsc1 is achieved through the CRD domain. Adhesion force maps (1 µm×1 µm) recorded on the surface of mutants Wsc1_C4,5A_ (a) Wsc1_C6,7A_ (b) and Wsc1_C8A_ (c), with a Ni^++^-NTA-tip in buffer solution at 25°C. While Wsc1 mutants showed a surface density similar to that of the wild-type, they were evenly distributed thus no longer clustered (see also Fig. 2c). The data shown are representative of results obtained on 17 different cells using 13 different tips.

To analyze the relationship between the structure of Wsc1 and its clustering properties, we then investigated the role of the CRD domain by mapping the distribution of Wsc1 in the Wsc1_C4,5A_, Wsc1_C6,7A_ and Wsc1_C8A_ mutants expressed form a *CEN/ARS* plasmid in a *wsc1* deletion and equipped with the elongation and His-tag as described above (Fig. 4). The three CRD mutants showed adhesion frequencies and adhesion values similar to those of the wild-type (Fig. S1). In addition, detailed analysis of the force curves revealed very similar spring behaviours for both wild-type and mutants, demonstrating that the sensor mechanical properties are not determined by the CRD domain (Fig. S2). This substantiates our earlier studies showing that glycosylation of the sensor at the Ser/Thr-rich region (STR) adds to the stiffness of the extracellular region and is required for its spring properties [14]. However, adhesion maps demonstrated major differences in lateral sensor organization, when CRD mutants were compared with the wild-type (Fig. 4): all mutant sensors appeared to be evenly distributed, rather than clustered (see also Fig. 2c for quantification). Note that for these determinations, the total number of sensors detected for a certain area did not significantly differ from that observed on the wild-type. The above finding demonstrates the important role of the CRD domain in sensor clustering.

### Fluorescence microscopy shows vacuolar accumulation of non-clustering sensors

**Figure 5:**
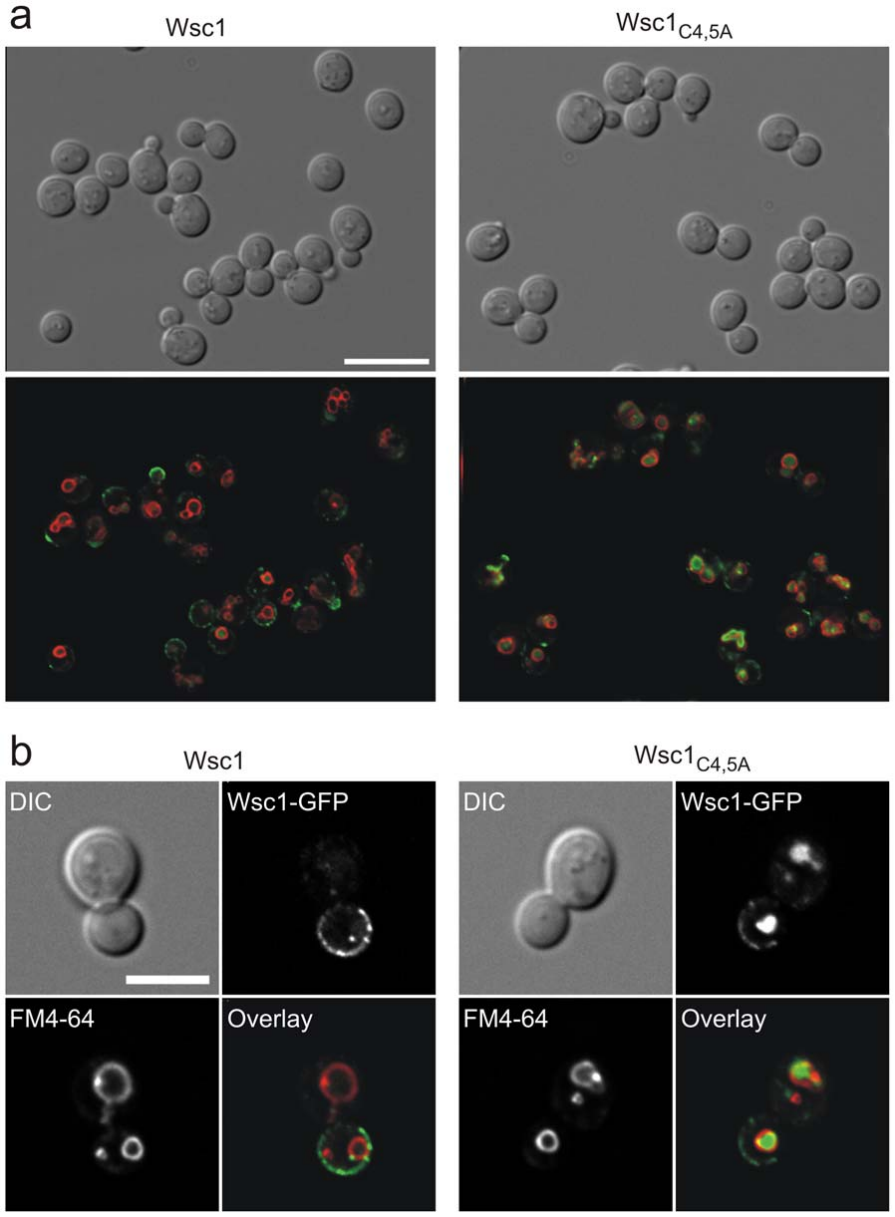
Fluorescence microscopy shows vacuolar accumulation of non-clustering sensors. (a) Elongated versions of Wsc1 and its mutant derivative Wsc1_C4,5A_ fused to GFP were expressed from a centromeric vector under the control of their native promoters in a *wsc1* deletion strain. A representative number of cells in different growth stages was examined by differential interference contrast (DIC) microscopy (upper row) and fluorescence microscopy (lower row). Wsc1-GFP signals are shown in green. The vacuolar membrane was stained with FM4-64 and is shown in red. Scale bar: 10 µm. (b) Larger image of cells in a late stage of cell division, expressing GFP fusions of either the wild-type Wsc1 or the Wsc1_C4,5A_ construct. Imaging conditions are as above, with the additional display of the separated signals from GFP and FM4-64 in the lower row. Scale bar: 5 µm.

As a complementary approach to our single-molecule experiments, we next used fluorescence microscopy with Wsc1-GFP proteins to visualize the distribution of the sensors in the plasma membrane of growing yeast cells (Fig. 5). As observed previously, Wsc1-GFP fusions show a dynamic distribution, with the signal concentrating at the emerging bud, then appearing within the cell and to some extent in the vacuole, before concentrating again at the bud-neck during cytokinesis [19]. This behaviour is also observed in the Wsc1_C4,5A_-GFP fusion. However, the latter is less frequently detected in plasma membrane spots, as compared to the wild-type Wsc1-GFP signal (Fig. 5b). Moreover, in general much higher signal intensities are observed in the vacuoles of the mutant strain as compared to the wild-type (Fig. 5a). It should be noted, that vacuolar signal intensities vary considerably between individual cells both in wild-type and mutant GFP fusions, explaining why cells with similar numbers of sensors on the surface could be chosen for the AFM measurements described above. A quantification of 45 budding cells from each culture yielded a mean signal intensity within the vacuoles of 79±31 arbitrary units for the wild-type Wsc1-GFP and 114±57 for Wsc1_C4,5A_-GFP.

## Discussion

Our current view of biological membranes emphasizes their high level of lateral compartmentation. In higher eukaryotes, lateral microdomains or lipid rafts enriched in sphingolipids and sterols have been speculated to favour segregation of specific membrane proteins like receptors or GPI-anchored proteins [3]. Membrane domains may have important roles in a variety of cellular functions including signalling, cell adhesion and membrane trafficking [4]. A prominent example are signalling processes, which in very diverse biological systems, from chemotaxis in *E. coli* [25] to the immune response in human T lymphocytes [26], frequently rely on - or are at least enhanced by - the local clustering of signalling components in or near microdomains of the plasma membrane. Because of the small size (<20–100 nm) and the highly dynamic nature of membrane domains in higher eukaryotes, their direct visualization in live cells remains very challenging, and consequently, their existence remains somewhat controversial [5], [27], [28]. In contrast, protein domains in yeast membranes have been shown to be sufficiently large and stable to be resolved by fluorescence microscopy [7]. Yet, the distribution, assembly and dynamics of single proteins within the domains have not been resolved owing to the lack of suitable probing techniques. Our experiments demonstrate that the combination of single-molecule AFM with genetic manipulation is a powerful platform for imaging protein clusters in living yeast cells in relation with function.

Single-molecule mapping revealed that most wild-type Wsc1 sensors concentrated within nanodomains of ∼200 nm diameter, which we tentatively call “sensosomes”. A pertinent question is whether the clustering we observed on the cell surface reflects similar clustering within the plasma membrane? We believe this is most likely the case, since we previously showed that the underlying mannosylated STR sequence adopts a rigid, nanospring structure [14], i.e. the flexibility between the CRD domain and the transmembrane domain (TMD) of the sensor should be very limited and provides a physical constraint to the lateral movement within the membrane. Since the nanospring mechanics of the sensor is not altered by the mutations of cysteine residues introduced in the CRD sequence (Fig. S2), this argument holds true for both wild-type and the mutant Wsc1 sensors studied here.

We showed that both the total amount of wild-type Wsc1 sensors and their frequency of clustering increased upon environmental stress, i.e. application of either heat or low osmolarity. Our finding is fully consistent with the notion that stress conditions activate the cell wall integrity pathway [29], and thus indicate that clustering is a stress-responsive process that is intimately connected to signalling. This behaviour is reminiscent of the tuning of signalling in bacterial chemotaxis, where clustering is known to dramatically increase the altitude of signal generation [25], [30]. We suggest that clustering is a means developed by yeast to concentrate Wsc1 sensors, and therefore the interacting downstream components, within a limited area to promote recruitment of the latter and thus enhance the cellular response. This mechanism is consistent with that of bacterial chemoreceptors, which form clusters of variable sizes (250±120 nm) that rearrange upon stimulation [31].

Since the incubation times under stress conditions employed here were only 15 min, the pronounced increase in Wsc1 density at the cell surface is hardly due to new protein synthesis. Rather, we suggest that the high intracellular dynamics of the sensor, as examplified by the rapid movement of Wsc1-GFP signals in time-lapse fluorescence microscopy [19], enables a fast increase of the plasma membrane-embedded fraction upon stress stimulation from internal stores. In addition, endocytosis of the plasma-membrane fraction of Wsc1 could be slowed-down in the clustered state, as deduced from the vast accumulation of Wsc1-GFP within the vacuoles of the CRD mutants. Note that an alternative explanation would be a specific endocytosis of inactive sensors independent from clustering, which cannot be ruled out by our data. Both processes therefore lead to a high local density of sensors within the plasma membrane under stress conditions, consequently enhancing the signal. In summary, these observations lead us to believe that Wsc1 clustering and signalling capacity are intimately correlated processes.

How is clustering of the sensors achieved? Our data clearly show that the CRD plays a crucial role in clustering, since mutation of the cysteines within this domain abolished both their clustering and their function *in vivo*. This substantiates previous observations, where the entire CRD region was deleted and shown to be crucial for sensor function [15]. The authors also observed that overproduction of Wsc1 inhibits cell growth, which could be explained in the light of our results from a constitutive activation of the CWI pathway by the increased sensor density. Overproduction of a CRD-less Wsc1 suppressed the phenotypes of a *wsc1* deletion, which may be attributed to an enhancement of the low sensor activity in the mutant by increasing its density. It should be noted that a substantial amount of wild-type Wsc1 sensor was shown to reside in detergent-resistant membrane fractions, i.e. in “lipid rafts” [15].

**Figure 6:**
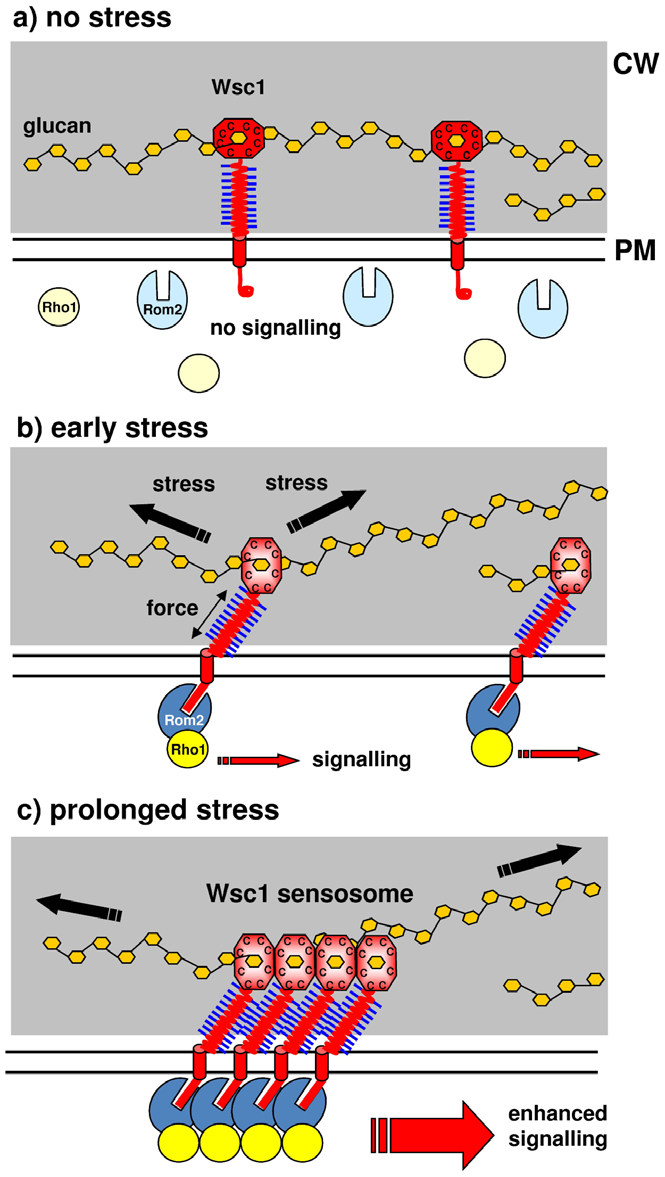
Biological significance of CRD-mediated Wsc1 clustering. (a) Non-stressing conditions. The CRD domains of Wsc1 sensors interact with cell wall glucans. Two representative glucan chains of the cell wall (CW, shaded in grey) are depicted as interconnected orange dots. Binding of the CRD domain should be transient to allow for lateral movement of sensors in the cell wall and plasma membrane. The CRD domain is followed by a Ser/Thr-rich (STR) region, with blue lines indicating mannosylation. The single transmembrane domain (TMD) of Wsc1 is shown as a red cylinder spanning the plasma membrane (PM). The cytoplasmic tail is curled and not competent for signalling to the thus inactive downstream components Rom2 and Rho1 (light blue and yellow, respectively). (b) As a first response to cell wall stress, the glucan chains are stretched, exerting a force on the Wsc1-STR nanospring [14] through their coupling to CRD. The induced conformational change in the cytoplasmic domain of the sensor, designated by the straight red bar, allows it to interact with and activate Rom2 (dark blue), which in turn promotes activation (exchange of GDP for GTP) of Rho1 (bright yellow), and signalling to the downstream components of the CWI pathway. Concomitantly, the CRD domain adapts an interaction-competent surface structure. (c) Intermolecular interactions of the CRD domains promote sensor clustering, with a concomitant increase of the downstream-signalling components at the internal side of the plasma membrane. This local accumulation enhances the stress signal and the cellular response. We propose to call this signalling complex a sensosome.

Because we have shown the key role of the CRD domain in mediating clustering, we believe that this domain may have broader functions than previously anticipated. Even though an X-ray structure is not available for any of the 86 CRD domains reported so far throughout all biological systems, a role of the domain in binding to carbohydrates has been suggested from its presence in a fungal exoglucanase [16]. Binding of Wsc1 to cell wall glucans through its CRD domain makes it ideally suited to function as a mechanosensor (Fig. 6): to detect stress acting on either the cell wall or the plasma membrane [10], [19], the sensor needs to be anchored in both structures, i.e. in the plasma membrane by the TMD sequence and in the cell wall by the CRD domain. Stress-induced deformation of the cell wall or membrane would then put a mechanical constraint (force) on the entire sensor. This in turn will likely lead to a conformational change in its cytoplasmic domain, which triggers the interaction with the downstream signalling components and thus induces the CWI response.

Carbohydrate binding however does not explain how the CRD domain mediates clustering of Wsc1. Moreover, a similar role of the CRD domain in carbohydrate-binding in the human mechanosensor polycystin seems rather redundant, since it also carries a number of PKD domains, which already fulfill this function [32]. As we have shown here that the cysteine mutants do not cluster anymore, we suggest an additional function for the CRD domain, that is to mediate protein-protein interactions. This would also provide an attractive model of how the large variety of environmental stresses reported to activate the CWI pathway [33] is sensed by Wsc1, as illustrated in Fig. 6. We propose that stretching of either the cell wall or the plasma membrane will alter the conformation of the CRD domain, thereby exposing interfaces promoting intermolecular protein-protein interactions and triggering the association of further sensor molecules within the plasma membrane. In analogy to bacterial two-component sensor kinases [30] and receptor tyrosine kinases in higher eukaryotes [34], this could alter the phosphorylation state of the Wsc1 cytoplasmic domain and thus its interaction with the following signalling component Rom2 [35].

In conclusion, our experiments demonstrate that single-molecule AFM provides new insights into the dynamic clustering of transmembrane sensors in *S. cerevisiae*. Our main findings are that i) the formation of Wsc1 nanoclusters is achieved via the CRD domain, ii) clustering is enhanced under stress conditions that activate the CWI signalling pathway, and iii) clustering and signalling capacity of Wsc1 are intimately correlated processes. This leads us to conclude that in yeast, like in higher eukaryotes, sensor function is coupled to a localized enrichment of sensors or receptors in membrane patches. We therefore expect our newly observed Wsc1 nanoclusters, i.e. the “sensosome”, to promote the concomitant accumulation of downstream signalling components of the CWI signalling pathway at the inner leavelet of the plasma membrane.

## Methods

### Media, growth conditions, strains and genetic manipulations

Strains HD56-5A (*MATα ura3-52 leu2-3,112 his3-11,15*) and HSK1.1 (*MATa wsc1::SpHIS5 mid2::loxP ura3-52 leu2-3,112 his3-11,15*) have already been described [36]. Strain HOD48-1D (*MATa wsc1::KlURA3 ura3-52 leu2-3,112 his3-11,15*) was obtained in the same genetic background, using the homozygous diploid DHD5 [37] as a recipient for transformation with a *KlURA3* deletion cassette generated by PCR with the oligonucleotides A10 and A11 (Table S1) from the template vector pUG72 [38]. The heterozygous deletion obtained was sporulated and subjected to tetrad analysis to yield the segregant HOD48-1D.

All sequences of the plasmids described in the subsequent paragraphs are available from J.J.H. upon request. Cysteine mutants were obtained by *in vitro* mutagenesis with the “QuickChange Site-Directed-Mutagenesis” kit from Stratagene (La Jolla, California) and the oligonucleotides 07.6-07.15 listed in Table S1. As a template for mutagenesis we used pSK1, a pUK1921 [39] derivative carrying the entire *WSC1* gene with its flanking sequences on a PstI fragment. Each mutant allele was then subcloned into the PstI site of the vector pUG6 [40], to place a selectable *kanMX4* marker downstream of *WSC1*. 3′ non-coding sequences of *WSC1* were then obtained by PCR with the oligonucleotides 05.149/05.150 and introduced into the single SacI site downstream of the *kanMX4* marker in each construct. From those, the *WSC1* alleles including the selection marker were excised with SalI/BssSI and used to substitute the *wsc1::SpHIS5* deletion in strain HSK1.1 by *in vivo* recombination. Thus, strains carrying the alleles *WSC1_C1A_*, *WSC1_C2,3A_*, *WSC1_C4,5A_*, *WSC1_C6,7A_*, and *WSC1_C8A_*, each at their native chromosomal locus, were obtained and designated HSK20-HSK24, respectively.

The construction of a hybrid vector encoding an elongated Wsc1-Mid2 sensor with a 8xHis tag for surface detection by AFM (pBH01) has been described in detail in [36]. Briefly, a *KlURA3* cassette introduced into the 5′ coding region of *WSC1* carried on a *CEN/ARS* plasmid with a YCplac111 [41] backbone was substituted by *in vivo* recombination for a PCR-generated fragment from *MID2*, with the His-tag sequence supplied by the 5′ oligonucleotide. For introduction of the mutated alleles, pBH01 was digested with BamHI/BstAPI and the backbone was ligated to a fragment carrying most of the coding sequence and the terminator of the *WSC1* cysteine mutants obtained from the pUG6 derivatives described above, to yield pJJH1169 (*WSC1_C4,5A_*), pSK401 (*WSC1_C6,7A_*) and pSK402 (*WSC1_C8A_*).

To obtain GFP fusions of the elongated constructs, pBH01 and pJJH1169 were each transformed into strain DHD5 in combination with a PCR fragment generated with the oligonucleotide pair 03.50/03.51 and the template vector pFA6a-GFP-kanMX4 [42]. After *in vivo* recombination and selection for G418, the plasmids were isolated from yeast transformants and amplified in *E. coli* [36]. They were called pJJH1191 (pBH01-GFP) and pJJH1192 (pJJH1169-GFP), respectively. For examination by fluorescence microscopy they were introduced into yeast strain HOD48-1D selecting for leucine prototrophy. Procedures and equipment for fluorescence microscopy have been explained in detail in [18].

For AFM studies, transformants were cultured in leucine-free synthetic media as follows. Two or three colonies from the solid medium plate used as inoculum were transferred into culture medium. Cells were agitated overnight at 30°C, grown up to the late logarithmic phase, and harvested by centrifugation. They were washed three times with sodium acetate buffer in buffered solutions (sodium acetate, pH 4.75), and resuspended in 10 mL buffer to a concentration of ∼10^6^ cells per mL.

### Atomic force microscopy

Gold-coated cantilevers were cleaned for 15 min by UV and ozone treatment, rinsed with ethanol, dried with a gentle nitrogen flow, immersed overnight in ethanol containing 0.01 mM of NTA-terminated alkanethiols (ProChimia, Poland), and then rinsed with ethanol. Unless stated otherwise, cantilevers were immersed in a 40 mM aqueous solution of NiSO_4_ (pH 7.2) for 1 h and rinsed with buffer before use. AFM measurements were performed in buffered solutions (sodium acetate +0.1 M sucrose, pH 4.75), using a Nanoscope IV Multimode AFM (Veeco Metrology Group, Santa Barbara, CA) and oxide sharpened microfabricated Si_3_N_4_ cantilevers (Olympus Ltd., Tokyo, Japan). Cells were immobilized by mechanical trapping into porous polycarbonate membranes (Millipore) [14]. The spring constants of the cantilevers were measured using the thermal noise method (Picoforce, Veeco Metrology Group), yielding values ranging from 0.02 to 0.028 N/m. Unless otherwise specified, all force measurements were performed using a constant approach and retraction speed of 1,500 nm/s, and with an interaction time of 500 ms. Adhesion maps were obtained by recording 32×32 force curves on 1 µm×1 µm areas of the cells, calculating the adhesion force values and displaying them as grey pixels.

## Supporting Information

Figure S1 Detection of single Wsc1 sensors. Adhesion force histogram (n = 4096) and representative force curves recorded with a Ni^++^-NTA-tip in buffer solution (sodium acetate + sucrose 100 mM + NiSO4 40 mM; pH 4.75) on the surface of the wild-type Wsc1 (a) and mutants Wsc1C4,5A (b), Wsc1C6,7A (c) and Wsc1C8A (d). All curves were obtained at 25°C using a retraction speed of 1,500 nm s-1 and an interaction time of 500 ms. The 207±54 pN mean adhesion forces document the detection of single His-tagged sensors.
(0.09 MB DOC)

Figure S2 Wild-type Wsc1 and CRD mutants show similar linear nanospring behaviors. Representative force-extension curves obtained upon stretching single wild-type Wsc1 (a), and mutants Wsc1C4,5A (b), Wsc1C6,7A (c) and Wsc1C8A (d). All curves display a linear region where force is directly proportional to extension, thus characteristic of a Hookean spring. Using the slope of the linear portion of the raw deflection vs. piezo displacement curves [14], we found that the spring constant ks of Wsc1 wild-types and mutants are all in the range of 4.3–4.6 pN nm-1.
(0.08 MB DOC)

Table S1 Oligonucleotides used in this work.
(0.04 MB DOC)

## References

[1] Linggi B, Carpenter G (2006). ErbB receptors: new insights on mechanisms and biology.. Trends Cell Biol.

[2] Smith-Garvin JE, Koretzky GA, Jordan MS (2009). T cell activation.. Annu Rev Immunol.

[3] Simons K, Ikonen E (1997). Functional rafts in cell membranes.. Nature.

[4] Michel V, Bakovic M (2007). Lipid rafts in health and disease.. Biol Cell.

[5] Jacobson K, Dietrich C (1999). Looking at lipid rafts?. Trends Cell Biol.

[6] Lingwood D, Simons K (2010). Lipid rafts as a membrane-organizing principle.. Science.

[7] Grossmann G, Opekarova M, Malinsky J, Weig-Meckl I, Tanner W (2007). Membrane potential governs lateral segregation of plasma membrane proteins and lipids in yeast.. EMBO J.

[8] Strádalová V, Stahlschmidt W, Grossmann G, Blazíková M, Rachel R (2009). Furrow-like invaginations of the yeast plasma membrane correspond to membrane compartment of Can1.. J Cell Sci.

[9] Klis FM, Boorsma A, De Groot PW (2006). Cell wall construction in *Saccharomyces cerevisiae*.. Yeast.

[10] Levin DE (2005). Cell wall integrity signaling in *Saccharomyces cerevisiae*.. Microbiol Mol Biol Rev.

[11] Rajavel M, Philip B, Buehrer BM, Errede B, Levin DE (1999). Mid2 is a putative sensor for cell integrity signaling in Saccharomyces cerevisiae.. Mol Cell Biol.

[12] Verna J, Lodder A, Lee K, Vagts A, Ballester RA (1997). family of genes required for maintenance of cell wall integrity and for the stress response in *Saccharomyces cerevisiae*.. Proc Natl Acad Sci U S A.

[13] Heinisch JJ, Lorberg A, Schmitz HP, Jacoby JJ (1999). The protein kinase C-mediated MAP kinase pathway involved in the maintenance of cellular integrity in *Saccharomyces cerevisiae*.. Mol Microbiol.

[14] Dupres V, Alsteens D, Wilk S, Hansen B, Heinisch JJ (2009). The yeast Wsc1 cell surface sensor behaves like a nanospring in vivo.. Nat Chem Biol.

[15] Lodder AL, Lee TK, Ballester R (1999). Characterization of the Wsc1 protein, a putative receptor in the stress response of *Saccharomyces cerevisiae*.. Genetics.

[16] Ponting CP, Hofmann K, Bork P (1999). A latrophilin/CL-1-like GPS domain in polycystin-1.. Curr Biol.

[17] Qian F, Wei W, Germino G, Oberhauser A (2005). The nanomechanics of polycystin-1 extracellular region.. J Biol Chem.

[18] Rodicio R, Buchwald U, Schmitz HP, Heinisch JJ (2008). Dissecting sensor functions in cell wall integrity signaling in *Kluyveromyces lactis*.. Fungal Genet Biol.

[19] Straede A, Heinisch JJ (2007). Functional analyses of the extra- and intracellular domains of the yeast cell wall integrity sensors Mid2 and Wsc1.. FEBS Lett.

[20] Muller DJ, Helenius J, Alsteens D, Dufrene YF (2009). Force probing surfaces of living cells to molecular resolution.. Nat Chem Biol.

[21] Puchner EM, Gaub HE (2009). Force and function: probing proteins with AFM-based force spectroscopy.. Curr Opin Struct Biol.

[22] Ahimou FO, Touhami A, Dufrêne YF (2003). Real-time imaging of the surface topography of living yeast cells by atomic force microscopy.. Yeast.

[23] Verbelen C, Gruber HJ, Dufrene YF (2007). The NTA-HiS(6) bond is strong enough for AFM single-molecular recognition studies.. J Mol Recognit.

[24] Lagerholm BC, Weinreb GE, Jacobson K, Thompson NL (2005). Detecting microdomains in intact cell membranes.. Annu Rev Phys Chem.

[25] Keymer JE, Endres RG, Skoge M, Meir Y, Wingreen NS (2006). Chemosensing in *Escherichia coli*: two regimes of two-state receptors.. Proc Natl Acad Sci U S A.

[26] Alonso MA, Millan J (2001). The role of lipid rafts in signalling and membrane trafficking in T lymphocytes.. J Cell Sci.

[27] Kenworthy AK (2008). Have we become overly reliant on lipid rafts? Talking Point on the involvement of lipid rafts in T-cell activation.. EMBO Rep.

[28] Munro S (2003). Lipid rafts: elusive or illusive?. Cell.

[29] Martin H, Rodriguez-Pachon JM, Ruiz C, Nombela C, Molina M (2000). Regulatory mechanisms for modulation of signaling through the cell integrity Slt2-mediated pathway in *Saccharomyces cerevisiae*.. J Biol Chem.

[30] Gestwicki JE, Kiessling LL (2002). Inter-receptor communication through arrays of bacterial chemoreceptors.. Nature.

[31] Besschetnova TY, Montefusco DJ, Asinas AE, Shrout AL, Antommattei FM (2008). Receptor density balances signal stimulation and attenuation in membrane-assembled complexes of bacterial chemotaxis signaling proteins.. Proc Natl Acad Sci U S A.

[32] Zelensky AN, Gready JE (2005). The C-type lectin-like domain superfamily.. FEBS J.

[33] Straede A, Corran A, Bundy J, Heinisch JJ (2007). The effect of tea tree oil and antifungal agents on a reporter for yeast cell integrity signalling.. Yeast.

[34] Huh KH, Fuhrer C (2002). Clustering of nicotinic acetylcholine receptors: from the neuromuscular junction to interneuronal synapses.. Mol Neurobiol.

[35] Vay HA, Philip B, Levin DE (2004). Mutational analysis of the cytoplasmic domain of the Wsc1 cell wall stress sensor.. Microbiology.

[36] Heinisch JJ, Dupres V, Alsteens D, Dufrene YF (2010). Measurement of the mechanical behaviour of yeast membrane sensors using single-molecule atomic force microscopy.. Nature Protocols.

[37] Arvanitidis A, Heinisch JJ (1994). Studies on the function of yeast phosphofructokinase subunits by in vitro mutagenesis.. J Biol Chem.

[38] Gueldener U, Heinisch J, Koehler GJ, Voss D, Hegemann JH (2002). A second set of loxP marker cassettes for Cre-mediated multiple gene knockouts in budding yeast.. Nucleic Acids Res.

[39] Heinisch JJ (1993). PFK2, ISP42, ERG2 and RAD14 are located on the right arm of chromosome XIII.. Yeast.

[40] Wach A, Brachat A, Alberti-Segui C, Rebischung C, Philippsen P (1997). Heterologous HIS3 marker and GFP reporter modules for PCR-targeting in *Saccharomyces cerevisiae*.. Yeast.

[41] Gietz RD, Sugino A (1988). New yeast-*Escherichia coli* shuttle vectors constructed with in vitro mutagenized yeast genes lacking six-base pair restriction sites.. Gene.

[42] Longtine MS, McKenzie A, Demarini DJ, Shah NG, Wach A (1998). Additional modules for versatile and economical PCR-based gene deletion and modification in *Saccharomyces cerevisiae*.. Yeast.

