# Supplementary material for: Single-Molecule Atomic Force Microscopy Reveals Clustering of the Yeast Plasma-Membrane Sensor Wsc1
Source: PLoS One. 2010 Jun 14;5(6):e11104. doi: 10.1371/journal.pone.0011104 (PMC2885430; doi:10.1371/journal.pone.0011104)
Supplement: Figure S1 — Detection of single Wsc1 sensors. Adhesion force histogram (n = 4096) and representative force curves recorded with a Ni++-NTA-tip in buffer solution (sodium acetate + sucrose 100 mM + NiSO4 40 mM; pH 4.75) on the surface of the wild-type Wsc1 (a) and mutants Wsc1C4,5A (b), Wsc1C6,7A (c) and Wsc1C8A (d). All curves were obtained at 25°C using a retraction speed of 1,500 nm s-1 and an interaction time of 500 ms. The 207±54 pN mean adhesion forces document the detection of single His-tagged sensors. (0.09 MB DOC) [file pone.0011104.s001.doc]

**Figure S1.** Detection of single Wsc1 sensors. Adhesion force histogram (n = 4096) and representative force curves recorded with a Ni^++^-NTA-tip in buffer solution (sodium acetate + sucrose 100 mM + NiSO_4_ 40 mM; pH 4.75) on the surface of the wild-type Wsc1 (a) and mutants Wsc1_C4,5A_ (b), Wsc1_C6,7A_ (c) and Wsc1_C8A_ (d). All curves were obtained at 25°C using a retraction speed of 1,500 nm s^-1^ and an interaction time of 500 ms. The 207 ± 54 pN mean adhesion forces document the detection of single His-tagged sensors.
