# Supplementary material for: Single-Molecule Atomic Force Microscopy Reveals Clustering of the Yeast Plasma-Membrane Sensor Wsc1
Source: PLoS One. 2010 Jun 14;5(6):e11104. doi: 10.1371/journal.pone.0011104 (PMC2885430; doi:10.1371/journal.pone.0011104)
Supplement: Figure S2 — Wild-type Wsc1 and CRD mutants show similar linear nanospring behaviors. Representative force-extension curves obtained upon stretching single wild-type Wsc1 (a), and mutants Wsc1C4,5A (b), Wsc1C6,7A (c) and Wsc1C8A (d). All curves display a linear region where force is directly proportional to extension, thus characteristic of a Hookean spring. Using the slope of the linear portion of the raw deflection vs. piezo displacement curves [14], we found that the spring constant ks of Wsc1 wild-types and mutants are all in the range of 4.3–4.6 pN nm-1. (0.08 MB DOC) [file pone.0011104.s002.doc]

**Figure S2.** Wild-type Wsc1 and CRD mutants show similar linear nanospring behaviors. Representative force-extension curves obtained upon stretching single wild-type Wsc1 (a), and mutants Wsc1_C4,5A_ (b), Wsc1_C6,7A_ (c) and Wsc1_C8A_ (d). All curves display a linear region where force is directly proportional to extension, thus characteristic of a Hookean spring. Using the slope of the linear portion of the raw deflection vs piezo displacement curves [14], we found that the spring constant *k_s_* of Wsc1 wild-types and mutants are all in the range of 4.3 - 4.6 pN nm^-1^.
