# Supplementary material for: Single-Molecule Atomic Force Microscopy Reveals Clustering of the Yeast Plasma-Membrane Sensor Wsc1
Source: PLoS One. 2010 Jun 14;5(6):e11104. doi: 10.1371/journal.pone.0011104 (PMC2885430; doi:10.1371/journal.pone.0011104)
Supplement: Table S1 — Oligonucleotides used in this work. (0.04 MB DOC) [file pone.0011104.s003.doc]

# Table S1: Oligonucleotides used in this work.

| **Number** | **Sequence (5´→ 3´)** |
| --- | --- |
| A10 | GGATAGTACGTTGGCTGATTTAGTACTCAGGATAAAAAATTCTATTTAAATAATGTTCGTACGCTGCAGGTCGAC |
| A11 | GGAACAAGACTTGCTTGGCAATAGTTTAAGAATATAATAATTTTTTTTGGGCATAGGCCACTAGTGGATCTG |
| 03.50 | ACCAGGAGGGAAAAACAACGTTTTAACAGTGGTCAATCCAGACGAAGCTGATCGGATCCCCGGGTTAATTAA |
| 03.51 | GGAGATGATTTGGCAAAATGAAATCGGAAAAAGAAAAAATTAATGGGAAGGAATTCGAGCTCGTTTAAAC |
| 05.149 | GCTGAGCTCAACATTAAATAGGAACAACAAAATTAG |
| 05.150 | GGTTGAGCTCGCCAGGACTTGAACCTGGAATC |
| 07.6 (C8A) | TGGTTACAGCAGTGAGATGgcaGGTGGTGAAGATGCATATTCTGTGTACCAAC |
| 07.7 (C8A) | GTTGGTACACAGAATATGCATCTTCACCACCtgcCATCTCACTGCTGTAACCA |
| 07.8 (C6,7A) | GTTCGGAATCTACTTCCTCTTCAgctAATACGTATgcaTTTGGTTACAGCAGTGAGATG |
| 07.9 (C6,7A) | CATCTCACTGCTGTAACCAAAtgcATACGTATTagcTGAAGAGGAAGTAGATTCCGAA |
| 07.10 (C4,5A) | CTTTGCCCTTTATAATCATTCAGAAgcaTATgctGGTGATACTAATCCATCTGGTTC |
| 07.11 (C4,5A) | GAACCAGATGGATTAGTATCACCagcATAtgcTTCTGAATGATTATAAAGGGCAAAG |
| 07.12 (C2,3A) | TAACTGGCAGTCGAGTTCACACgcaAACAGTGAGgctAGCGCAAAAGGTGCAAGCTAC |
| 07.13 (C2,3A) | GTAGCTTGCACCTTTTGCGCTagcCTCACTGTTtgcGTGTGAACTCGACTGCCAGTTA |
| 07.14 (C1A) | AGCGAACGCCTATGAATACGTGAATgcaTTTAGCTCACTACCCTCTGACTTTTC |
| 07.15 (C1A) | GAAAAGTCAGAGGGTAGTGAGCTAAAtgcATTCACGTATTCATAGGCGTTCGCTT |

Information in parentheses after the oligonucleotide number indicate the cysteine mutants obtained with them. Restriction sites used for cloning are underlined; base exchanges for *in vitro* mutagenesis are depicted in small print.
